# Early 60-Day Morbidity after Sleeve Gastrectomy Versus One-Anastomosis Gastric Bypass: A Propensity-Matched Single-Center Cohort of 2,382 Patients

**DOI:** 10.1007/s11695-026-08650-0

**Published:** 2026-04-15

**Authors:** Ohad Guetta, Illia Vasyliev, Anton Osyntsov, Alex Vakhrushev, Sharon Daniel, Yuval Arnon, Oleg Dukhno

**Affiliations:** 1 Department of General Surgery B, Soroka Medical Center, Be’er Sheva, Israel; 2 The Faculty of Health Sciences, Ben-Gurion University of the Negev, Be’er Sheva, Israel; 3 Department of Pediatric Surgery, Soroka Medical Center, Be’er Sheva, Israel; 4 Department of Epidemiology, Biostatistics, and Community Health Sciences, School of Public Health, Faculty of Health Sciences, Ben-Gurion University of the Negev, Be’er Sheva, Israel; 5 Clinical Research Center, Soroka University Medical Center and Faculty of Health Sciences, Ben-Gurion University of the Negev, Be’er Sheva, Israel

**Keywords:** Sleeve Gastrectomy, One-anastomosis gastric bypass, Complications, Clavien-Dindo classification, Leak, morbid obesity

## Abstract

**Background:**

Comparative data on early complications after sleeve gastrectomy (SG) versus one-anastomosis gastric bypass (OAGB) remain limited.

**Objective:**

To compare 60-day postoperative morbidity between SG and OAGB while accounting for confounding.

**Setting:**

Soroka University Medical Center, Israel.

**Methods:**

This is a retrospective cohort study assessing 60-day morbidity in patients undergoing SG and OAGB between 2009 and 2024. Propensity scores (sex, height, BMI, number of previous bariatric surgeries, smoking status, diabetes mellitus, fatty liver, hiatal hernia, cholecystectomy during index surgery, and band removal during index surgery) were used for 1:1 nearest-neighbor matching without replacement. Any potential postoperative events were flagged by utilization triggers (length of stay > 7 days, ICU admission, blood transfusion, CT or upper endoscopy within 60 days) and verified by chart review. The primary outcome of this study was any complication occurring ≤ 60 days from the operation; secondary outcomes included complication types and severity by Clavien-Dindo classification.

**Results:**

Of 3,317 patients (SG *n* = 1,816; OAGB *n* = 1,501), 1,191 matched pairs were analyzed. Overall, 60-day complications were higher after SG than OAGB (15.4% vs. 11.8%, *p* = 0.012). SG patients exhibited more Clavien–Dindo grade II (5.1% vs. 2.4%, *p* > 0.001), IIIa events (1.3% vs. 0.3%, *p* = 0.003) and IVb events (0.7% vs. 0.1%, *p* = 0.039). SG patients experienced higher rates of leak (1.8% vs. 0.8%, *p* = 0.019), abscess (1.2% vs. 0%, *p* < 0.001), pleural and abdominal fluid collection (1.2% vs. 0.3%, *p* = 0.007), bleeding (1.6% vs. 0.7%, *p* = 0.033) and dysphagia/vomiting (3.2% vs. 1.3%, *p* = 0.001) compared with OAGB patients. OAGB patients presented with more endoscopically diagnosed ulcers (0.7% vs. 0%, *p* = 0.008) and respiratory complaints (2.2% vs. 0.6%, *p* < 0.001) compared to SG patients. Among complicated patients, upper endoscopy within 60 days was more frequent after SG (29.3% vs. 17.3%, *p* = 0.005); ICU length of stay was not different between the groups (0.8 ± 5.4 days in OAGB vs. 1.2 ± 9.1 days in SG, *p* = 0.5).

**Conclusions:**

In a large propensity-matched single-center cohort study, SG showed higher 60-day morbidity than OAGB, primarily of intermediate severity. Limitations include retrospective design, temporal bias, trigger-based ascertainment, and lack of capture of care delivered at external hospitals.

**Supplementary Information:**

The online version contains supplementary material available at 10.1007/s11695-026-08650-0.

## Key points

In 2,382 patients, SG had higher overall 60-day complications than OAGB after matching.

Differences were mostly driven by CD II, IIIa and IVb events.

Severe complications (CD IIIb–V) were low and comparable.

Propensity matching mitigates selection bias.

**Supplementary Information:**

The online version contains supplementary material available at 10.1007/s11695-026-08650-0.

## Introduction

Obesity is a leading global health problem and has reached epidemic proportions, with more than 890 million adults and 160 million children affected worldwide according to the World Health Organization, and projections estimating more than 1.5 billion adults with obesity by 2035 [1]. Bariatric surgery has therefore risen as one of the most reliable, effective, and durable treatments for severe obesity and its comorbidities, with nearly 600,000 procedures performed globally in 2021 [2–5]. These numbers reflect the low utilization of bariatric surgery compared to the increased prevalence of the disease [6], with only ~ 1–2% of eligible patients undergo surgery annually, in part due to access limitations, cost, eligibility criteria, and the potential for postoperative complications. Sleeve gastrectomy (SG) and Roux-en-Y gastric bypass (RYGB) are still the two most frequently performed bariatric procedures worldwide [1], followed by one-anastomosis gastric bypass (OAGB, also termed single-anastomosis, mini- or omega-loop gastric bypass). While both SG and OAGB achieve excellent weight loss and metabolic improvement, their short-term complication profiles differ due to anatomical and physiological features. SG predominantly involves a long gastric staple line and is thus prone to staple-line–related complications such as bleeding, leak, or stricture. In contrast, OAGB introduces a gastrojejunostomy, which carries additional risks of marginal ulcer, anastomotic leak, bile reflux, or obstruction [7–9]. Previous registry analyses and randomized trials mainly compared SG with RYGB [4, 10] while head-to-head evidence specifically between SG and OAGB remains limited. An international propensity-matched study (GENEVA) reported comparable 30-day morbidity between SG and OAGB. However, single-center data with standardized complication assessment and extended follow-up remain limited, justifying further focused evaluation [11]. We therefore aimed to compare early postoperative outcomes of SG and OAGB in a large single-center cohort. Propensity score matching methods were utilized to minimize confounding by indication, reducing confounding bias.

## Methods

We conducted a retrospective cohort study at Soroka University Medical Center (SUMC). SUMC is a tertiary referral 1,100-beds hospital in southern Israel that serves a catchment population of approximately one million residents and provides comprehensive surgical, radiological (including interventional), and intensive care services. The study was approved by the SUMC Institutional Review Board.

### Data Collection

Demographic variables (sex and age) and anthropometric measures (weight, height, and BMI) were extracted from each patient’s electronic medical record. Information on chronic conditions was abstracted from primary care digital records and/or index-admission documentation. The following covariates were recorded: smoking status, ischemic heart disease, congestive heart failure, cerebrovascular disease (CVA), hypertension, chronic renal failure, chronic obstructive pulmonary disease (COPD), diabetes mellitus, malignancy, hiatal hernia, prescription of a proton pump inhibitor (PPI) in the year preceding surgery, fatty liver, hyperlipidemia, obstructive sleep apnea (OSA), orthopedic disorders (any chronic condition of the spine, back, pelvis, or knee), vitamin B12 deficiency, iron deficiency, and hypothyroidism. Surgical data included operative duration, previous bariatric procedures, concomitant cholecystectomy, and concomitant gastric band removal.

To identify potential postoperative events, we applied the following utilization-based triggers: hospital length of stay > 7 days, admission to the intensive care unit, receipt of blood transfusion, and performance of a CT scan or upper gastrointestinal endoscopy within 60 days after surgery. For every patient meeting at least one trigger, the complete chart was reviewed to confirm and classify complications. Our decision to include events in the first 60 days after surgery, rather than the conventional 30 days reporting, stems from the will to include milder complications that may affect the postoperative course and quality of life of the patient.

### Outcomes

Complications were defined as adverse events occurring within 60 days of surgery, identified through chart review and validated by imaging, laboratory, or re-intervention when applicable. Severity was graded using the Clavien–Dindo classification (CD I–V) [12]. Specific complications analyzed included:

- Leak – Dehiscence of an anastomosis or staple line with clinical peritonitis requiring surgical or endoscopic intervention.
- Abscess – Deep surgical site infection necessitating percutaneous drainage and/or prolonged antibiotic therapy.
- Pleural or abdominal fluid collection – Fluid collection within the pleural or peritoneal cavity not requiring intervention.
- Ulcer – Gastric or gastrojejunal ulcer diagnosed by upper endoscopy.
- Bleeding – Clinically significant hemorrhage requiring monitoring, blood transfusion, and/or endoscopic or surgical intervention.
- Hematoma – Postoperative hematoma not requiring intervention.
- Abdominal pain – Postoperative abdominal pain managed with fluids and analgesia alone.
- Stricture or obstruction – Stricture (most commonly after sleeve gastrectomy), gastric volvulus, or small-bowel obstruction requiring endoscopic or surgical intervention.
- Dysphagia – Inability to tolerate semisolid or solid food requiring intravenous nutritional support.
- Dehydration – Inability to tolerate oral fluids postoperatively requiring admission for intravenous fluid replacement.
- Renal failure – Acute kidney injury not attributable to sepsis, hemorrhage, or dehydration.
- Gallstone disease – Biliary colic, cholecystitis, choledocholithiasis, or gallstone pancreatitis occurring within 60 days after surgery.
- Respiratory failure – Need for invasive or noninvasive ventilatory support.
- Respiratory complaints – Exacerbation of asthma or COPD, cough, or dyspnea.
- Hernia – Hernia requiring surgery, most commonly at trocar sites.
- Other infection – Pneumonia, bacteremia, tonsillitis, gastroenteritis, urinary tract infection, wound infection, cellulitis, etc.
- Myocardial infarction and heart failure.
- Other – Diagnoses such as Wernicke–Korsakoff syndrome, deep vein thrombosis, pancreatitis, chest pain, tachycardia, headache, or dizziness.
- Observation – Admissions for observation for causes not encompassed by the categories above.
- Death – Death attributable to surgery.

### Statistical Analysis

Baseline characteristics were compared using χ² or Fisher’s exact test for categorical variables and t-tests for continuous variables.

We used propensity score matching (PSM) to reduce selection bias and balance baseline differences between the SG and OAGB groups. PSM was used because the two surgical groups differed in several baseline clinical characteristics, reflecting the non-randomized nature of the cohort and the potential for selection bias. In observational studies, treatment allocation, here, the choice of SG vs. OAGB, is influenced by patient and clinical factors that may also affect postoperative outcomes. Propensity scores summarize these covariates into a single probability of receiving a given treatment, allowing patients with similar likelihoods of undergoing each procedure to be matched. This creates groups that are more comparable and reduces confounding by indication.

PSM was generated using logistic regression including the following variables: sex, height, BMI, number of previous bariatric surgeries, smoking status, diabetes mellitus, fatty liver, hiatal hernia, cholecystectomy during index surgery, and band removal during index surgery. One-to-one nearest-neighbor matching without replacement was then applied to construct two cohorts with balanced baseline characteristics, thereby strengthening the validity of the subsequent outcome comparisons.

Outcomes were analyzed in the matched cohort; two-sided *p* < 0.05 was considered statistically significant. All statistical analyses were performed using Python (version 3.10.12) with the packages *pandas* (v1.5.3), *statsmodels* (v0.13.5), *scipy* (v1.10.1), and *scikit-learn* (v1.2.2).

## Results

From 2009 to 2024, a total of 3,317 patients underwent SG (*n* = 1,816) or OAGB (*n* = 1,501). The general characteristics of both groups are represented in Table 1.

**Table 1:** General characteristics of sleeve gastrectomy and one-anastomosis gastric bypass groups

Variable	SG (*n* = 1,816)	OAGB (*n* = 1,501)	*p*-value
Age (years)	38.42 ± 12.73	39.02 ± 12.10	0.162
Weight (kg)	117.98 ± 21.57	116.69 ± 20.25	0.095
Height (cm)	166.08 ± 9.40	163.97 ± 8.63	< 0.001
BMI (kg/m^2^)	42.86 ± 6.32	43.47 ± 6.19	0.009
Sex (Female)	1211 (66.7%)	1099 (73.2%)	< 0.001
Smoking status	764 (42.1%)	547 (36.4%)	0.001
Ischemic heart disease	101 (5.6%)	106 (7.1%)	0.083
Congestive heart failure	25 (1.4%)	13 (0.9%)	0.2
CVA	52 (2.9%)	51 (3.4%)	0.4
Hypertension	513 (28.2%)	394 (26.2%)	0.210
Chronic renal failure	4 (0.2%)	0 (0.0%)	0.132
COPD	255 (14.0%)	273 (18.2%)	0.001
Diabetes mellitus	442 (24.3%)	497 (33.1%)	< 0.001
Malignancy	51 (2.8%)	51 (3.4%)	0.363
Hiatal Hernia	87 (4.8%)	130 (8.7%)	< 0.001
Prescribed PPI in the year before operation	330 (18.2%)	358 (23.9%)	< 0.001
Fatty Liver	367 (20.2%)	574 (38.2%)	< 0.001
Hyperlipidemia	492 (27.1%)	444 (29.6%)	0.121
OSA	121 (6.7%)	122 (8.1%)	0.109
Orthopedic disorders	938 (51.7%)	999 (66.6%)	< 0.001
B12 deficiency	177 (9.7%)	233 (15.5%)	< 0.001
Iron deficiency	260 (14.3%)	358 (23.9%)	< 0.001
Hypothyroidism	164 (9.0%)	141 (9.4%)	0.718
**Surgical Information**			
Procedure duration (Minutes)	72.9 ± 25.4	77.5 ± 30.2	< 0.001
Previous bariatric surgeries	454 (25.0%)	463 (30.8%)	< 0.001
Gastric band removal during index surgery	363 (20.0%)	372 (24.8%)	0.001
Cholecystectomy before index surgery	88 (4.8%)	82 (5.5%)	0.430
Cholecystectomy during index surgery	81 (4.5%)	52 (3.5%)	0.155

**Fig. 1:**
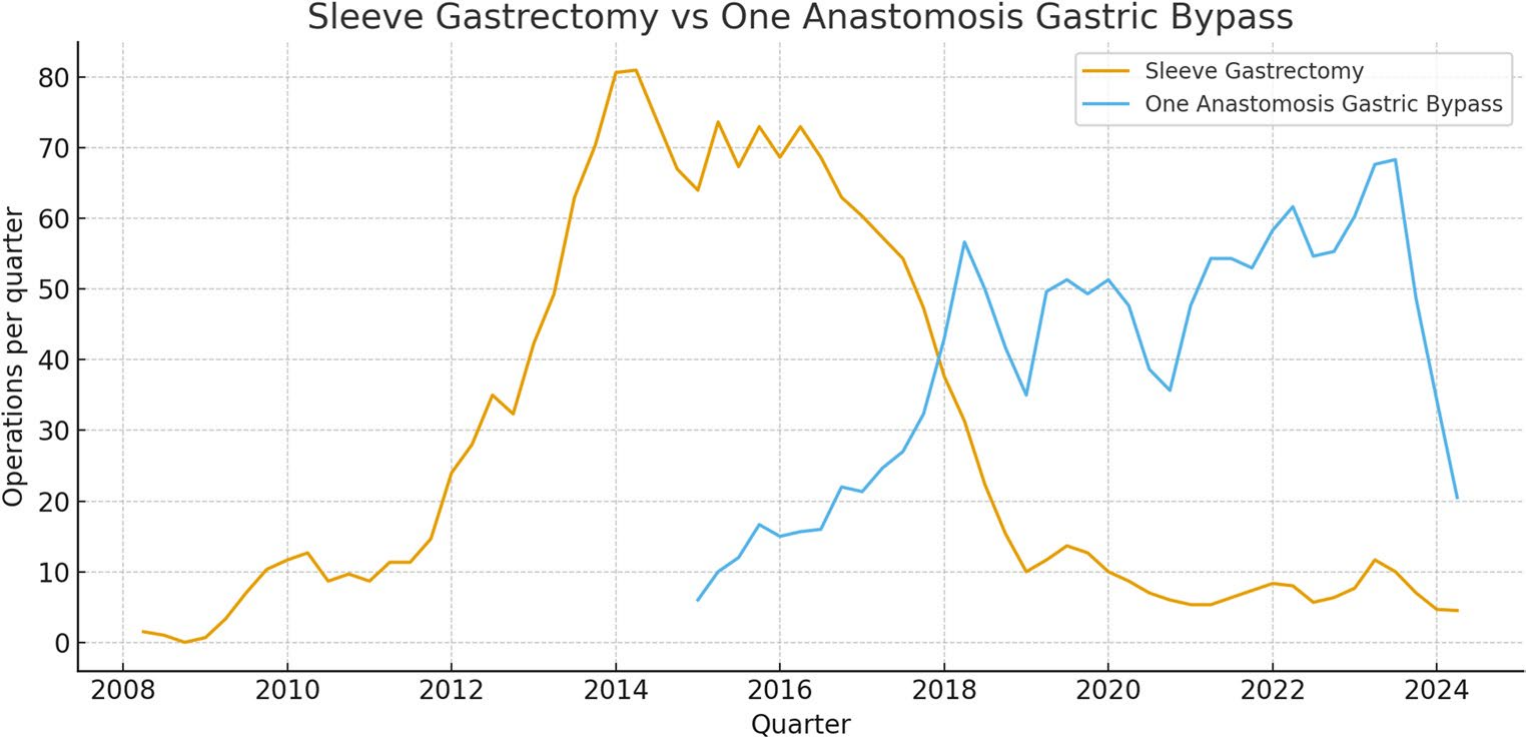
Frequency of bariatric surgery in SUMC between 2008-2024

We observed notable treatment-selection bias in our cohort, driven primarily by temporal shifts in procedure preference. From 2008 to 2015, SG predominated; from 2015 to the present, OAGB has been performed more frequently, reflecting evolving evidence regarding the relative benefits and risks of each operation (Fig. 1).

Consequently, baseline characteristics differed significantly between groups. To mitigate confounding by indication, we conducted propensity score matching, yielding two balanced cohorts of 1,191 patients each; the matched results are presented in Table 2.

**Table 2:** Propensity score matching balanced upon the following variables

Variable	SG (*n* = 1,191)	OAGB (*n* = 1,191)	*p*-value
Age (years)	38.3 ± 12.8	38.7 ± 12.1	0.5
Height (cm)	164.3 ± 8.8	164.4 ± 8.7	0.7
BMI (kg/m²)	43.1 ± 6.1	43.3 ± 6.5	0.5
A previous bariatric surgery	341 (28.6%)	356 (29.9%)	0.4
Gender (Female %)	882 (74.1%)	866 (72.7%)	0.5
Smoking status (%)	464 (39.0%)	458 (38.5%)	0.8
Diabetes mellitus (%)	335 (28.1%)	373 (31.3%)	0.1
Fatty liver (%)	325 (27.3%)	398 (33.4%)	0.001
Hiatal hernia (%)	74 (6.2%)	92 (7.7%)	0.2
Cholecystectomy during index surgery	41 (3.4%)	44 (3.7%)	0.8
Band removal during index surgery	266 (22.3%)	276 (23.2%)	0.7

### Complications by Type

Overall, 60-day complications were higher after SG than OAGB (15.4% vs. 11.8%, *p* = 0.012). SG patients experienced higher rates of leak (1.8% vs. 0.8%, *p* = 0.019), abscess (1.2% vs. 0%, *p* < 0.001), pleural and abdominal fluid collection (1.2% vs. 0.3%, *p* = 0.007), bleeding (1.6% vs. 0.7%, *p* = 0.033) and dysphagia/vomiting (3.2% vs. 1.3%, *p* = 0.001), compared with OAGB patients. OAGB patients presented with more endoscopically diagnosed ulcers (0.7% vs. 0%, *p* = 0.008) and respiratory complaints (2.2% vs. 0.6%, *p* < 0.001) compared to SG patients (Table 3). Out of 324 events of complications in the whole cohort, 49 were observed in the 30-to-60 days after operation (15.1%). No deaths occurred after SG, compared with one case (0.1%) after OAGB (*p* > 0.9, Fisher’s exact test). Although not statistically significant, this event reflects limited power, not true equivalence and warrants cautious interpretation given its clinical relevance.

**Table 3:** 60 days postoperative complications by type

Complication	SG (*n* = 1,191)	OAGB (*n* = 1,191)	*p*-value
Any complication	183 (15.4%)	141 (11.7%)	0.012
Leak	22 (1.5%)	9 (0.8%)	0.019
Abscess	14 (1.2%)	0 (0%)	< 0.001
Pleural/abdominal fluid	14 (1.2%)	3 (0.3%)	0.007
Ulcer	0 (0%)	8 (0.6%)	0.008
Bleeding	19 (1.6%)	8 (0.7%)	0.033
Hematoma	3 (0.3%)	6 (0.5%)	0.5
Abdominal pain	18 (1.5%)	28 (2.4%)	0.14
Stricture or obstruction	12 (1.0%)	5 (0.4%)	0.088
Dysphagia	38 (3.2%)	15 (1.3%)	0.001
Dehydration	4 (0.3%)	0 (0%)	0.12
Renal failure	1 (0.1%)	0 (0%)	> 0.9
Gallstone disease	4 (0.3%)	0 (0%)	0.2
Respiratory failure	2 (0.2%)	4 (0.3%)	0.7
Respiratory complaint	7 (0.6%)	26 (2.2%)	< 0.001
Hernia	5 (0.4%)	3 (0.3%)	0.7
Other infection	2 (0.2%)	1 (0.1%)	> 0.9
MI and Heart failure	0 (0%)	2 (0.1%)	0.5
Other	17 (1.4%)	17 (1.4%)	> 0.9
Observation	2 (0.2%)	3 (0.3%)	> 0.9
Death	0 (0.0%)	1 (0.1%)	> 0.9

### Complications by Severity

According to Clavien–Dindo classification, SG had more CD II (5.1% vs. 2.4%, *p* < 0.001), CD IIIa (1.3% vs. 0.3%, *p* = 0.003) and CD IVb (0.7% vs. 0.1%, *p* = 0.039) complications (Table 4). When classes IIIB to V were grouped together to severe complications - the difference was not significant (4.0% in SG vs. 2.7% in OAGB, *p* = 0.069).

**Table 4:** 60 days post operative complications by CD

CD grade	SG (*n* = 1,191)	OAGB (*n* = 1,191)	*p*-value
Any complication (CD Yes/No)	183 (15.4%)	141 (11.7%)	0.012
CD I	58 (4.9%)	78 (6.5%)	0.077
CD II	61 (5.1%)	28 (2.4%)	< 0.001
CD IIIa	16 (1.3%)	3 (0.3%)	0.003
CD IIIb	33 (2.8%)	21 (1.8%)	0.10
CD IVa	7 (0.6%)	9 (0.8%)	0.6
CD IVb	8 (0.7%)	1 (0.1%)	0.039
CD V	0 (0.0%)	1 (0.1%)	> 0.9

### Outcomes among Complicated Patients

Among patients who experienced complications, SG was associated with more upper endoscopy and duration of hospitalization. No significant difference was observed in the POD of complication, length of stay in hospital or ICU, CT performed, blood transfusions or reoperation (Table 5).

**Table 5:** Other outcomes of groups of complicated cases of SG and OAGB

Outcome	SG (*n* = 183)	OAGB (*n* = 141)	*p*-value
POD of complication	13.1 ± 15.4	11.4 ± 15.4	0.12
Length of stay	8.6 ± 14.0	6.2 ± 7.6	< 0.001
Upper endoscopy within 60 days	54 (29.5%)	22 (15.6%)	< 0.005
CT scan within 60 days	1.0 ± 1.4	1.0 ± 1.2	0.4
Packed cells transfused	0.155 ± 0.74	0.201 ± 0.80	0.615
Reoperations within 60 days	38 (20.8%)	26 (18.4%)	0.683
ICU admission	15 (5.0%)	16 (11.3%)	0.44

## Discussion

In this single-center cohort, SG was associated with a statistically higher 60-day complication rate than OAGB after propensity score matching (15.4% vs. 11.7%; *p* = 0.012), with SG showing more leak, dysphagia/vomiting, abscesses and pleural/abdominal fluid collections - patterns consistent with the known staple-line and functional risks after SG [13–17]. In contrast, OAGB was associated with more endoscopically diagnosed ulcers and respiratory complaints, in line with literature on marginal ulcers and reflux after bypass configurations (including OAGB) [10, 18–22]. Severe complications (CD IIIb–V) and mortality were uncommon and did not differ between procedures; this echoes international analyses (e.g., GENEVA) showing comparable early serious morbidity across SG, RYGB, and OAGB after adjustment [11].

Several considerations temper firm causal interpretation. First, despite matching, unmeasured confounding (e.g., PPI adherence, pulmonary disease severity, surgeon threshold) may persist. Second, data collection excluded encounters at external institutions. Consequently, patients operated on at SUMC but managed for complications elsewhere would not be captured in our dataset. Third, complication ascertainment relied on utilization-based triggers (upper endoscopy, CT within 60 days, prolonged length of stay, ICU admission) that may differ by procedure and clinician threshold, potentially influencing which events were detected and how they were classified. Fourth, a potential temporal bias exists, as SG and OAGB were performed during different periods in the surgical adoption timeline. Because the two procedures had minimal overlap in calendar years, we were unable to adjust for year of surgery or conduct time-restricted sensitivity analyses. Consequently, differences in outcomes may partly reflect evolving perioperative practices rather than the intrinsic characteristics of the procedures themselves. Fifth, bile reflux is a significant complication (mostly chronic and less early) of bariatric surgery with non-conclusive data in the literature [23]. This study, although showing higher rates of ulcers and respiratory complaints in the OAGB group (indirectly signals of bile reflux in that group), does not address this issue.

Also, hiatal hernia was significantly more common among patients undergoing OAGB before matching, suggesting a potential selection bias in surgical decision-making. This pattern likely reflects a clinical preference at our center to offer OAGB—often accompanied by concomitant hiatal hernia repair—to patients with known hernias. Although propensity score matching was applied to mitigate such baseline differences, residual bias cannot be entirely excluded. To address this concern, we conducted a sensitivity analysis excluding all patients with hiatal hernia, and achieved consistent results (Supplementary Table 1). Finally, multiple comparisons across numerous endpoints raise the likelihood of chance findings; p-values near 0.05 should be regarded as signals pending correction or external validation. In contrast to that, the CD categorization reflects the real clinical significance of our data.

Future work should incorporate calendar time and surgeon effects into the causal framework. In addition, the statistical analysis would have been strengthened by pre-specifying a limited set of clinically salient endpoints and applying multiplicity control.

In conclusion, this study shows that SG and OAGB may differ in the panel of mild complications’ rate (CD I to CD IIIa), but the more severe complications (CD IIIb to CD V) are rare and comparable between the procedures. This safety data about the procedures should support the utilization of bariatric surgery for the increasing epidemic of obesity.

## Supplementary Information

Below is the link to the electronic supplementary material.

ESM 1 (DOCX 13.6 KB)

## Data Availability

Guetta, Ohad (2025), “Data set of Bariatric surgeries - Sleeve gastrectomy and OAGB - short term complications analysis”, Mendeley Data, V1, doi: 10.17632/gb5r92cp3d.1.
