# Supplementary material for: Early 60-Day Morbidity after Sleeve Gastrectomy Versus One-Anastomosis Gastric Bypass: A Propensity-Matched Single-Center Cohort of 2,382 Patients
Source: Obes Surg. 2026 Apr 15;36(5):2458–64. doi: 10.1007/s11695-026-08650-0 (PMC13222240; doi:10.1007/s11695-026-08650-0)
Supplement: Supplementary file 1 — (DOCX 13.6 KB) [file 11695_2026_8650_MOESM1_ESM.docx]

**Supplementary Table 1: 60 days postoperative complications by type, excluding**

| **Complication** | **SG**  **(N = 1,121^1^)** | **OAGB**  **(N = 1,095^1^)** | **p-value** |
| --- | --- | --- | --- |
| **Any Complication** | 167 (15%) | 129 (12%) | 0.031 |
| **Leak** | 19 (1.7%) | 8 (0.7%) | 0.039 |
| **Abscess** | 12 (1.1%) | 1 (< 0.1%) | 0.003 |
| **Pleural/Abdominal Fluid** | 12 (1.1%) | 4 (0.4%) | 0.050 |
| **Ulcer** | 0 (0%) | 7 (0.6%) | 0.007 |
| **Bleeding** | 17 (1.5%) | 8 (0.7%) | 0.080 |
| **Hematoma** | 2 (0.2%) | 5 (0.5%) | 0.300 |
| **Abdominal Pain** | 15 (1.3%) | 27 (2.5%) | 0.052 |
| **Stricture/Obstruction** | 11 (1.0%) | 5 (0.5%) | 0.140 |
| **Dysphagia** | 39 (3.5%) | 14 (1.3%) | <0.001 |
| **Dehydration** | 3 (0.3%) | 0 (0%) | 0.200 |
| **Renal Failure** | 3 (0.3%) | 0 (0%) | 0.200 |
| **Gallstone Disease** | 1 (< 0.1%) | 1 (< 0.1%) | >0.9 |
| **Respiratory Failure** | 1 (< 0.1%) | 5 (0.5%) | 0.120 |
| **Respiratory Complaint** | 7 (0.6%) | 20 (1.8%) | 0.010 |
| **Hernia** | 4 (0.4%) | 3 (0.3%) | >0.9 |
| **Other Infection** | 0 (0%) | 1 (< 0.1%) | 0.500 |
| **MI / Heart Failure** | 0 (0%) | 2 (0.2%) | 0.200 |
| **Other** | 18 (1.6%) | 15 (1.4%) | 0.600 |
| **Observation** | 2 (0.2%) | 1 (< 0.1%) | >0.9 |
| **Death** | 1 (< 0.1%) | 1 (< 0.1%) | >0.9 |

**SG: Sleeve Gastrectomy**

**OAGB: One-Anastomosis Gastric Bypass**

| ^1^n (%) |
| --- |
| ^2^Pearson's Chi-squared test; Fisher's exact test |
